# The role of the zinc finger protein ZC3H32 in bloodstream-form *Trypanosoma brucei*

**DOI:** 10.1371/journal.pone.0177901

**Published:** 2017-05-17

**Authors:** Cornelia Klein, Monica Terrao, Christine Clayton

**Affiliations:** Centre for Molecular Biology of Heidelberg University, DKFZ-ZMBH Alliance, Heidelberg, Germany; University of Texas Medical School at Houston, UNITED STATES

## Abstract

Kinetoplastids rely heavily on post-transcriptional mechanisms for control of gene expression, with regulation of mRNA processing, translation and degradation by RNA-binding proteins. ZC3H32 is a cytosolic mRNA-binding protein with three non-canonical CCCH zinc finger domains. It is much more abundant in bloodstream-form *Trypanosoma brucei* than in procyclic forms. Tethering of ZC3H32 to a reporter mRNA suppressed translation and resulted in mRNA degradation, and deletion analysis suggested that this activity was present in both the N- and C-terminal domains, but not the central zinc finger-containing domain. Tandem affinity purification, however, revealed no interaction partners that might account for this activity. RNASeq analyses did not yield any evidence for sequence-specific binding or regulation of specific mRNAs. The presence of ZC3H32 homologues in monogenetic and free-living Euglenids also argues against a role in developmental regulation, although its function may have diverged in evolution. *T*. *brucei* ZC3H32 might be implicated in basal mRNA metabolism, with this role perhaps being taken over by another protein in procyclic forms.

## Introduction

Organisms in the Phylum Euglenozoa rely on *trans* splicing to process mRNAs from polycistronically transcribed precursors. This has been most extensively analysed in the parasitic trypanosomes and leishmanias, in which it has been shown that regulation of individual mRNAs relies heavily on post-transcriptional mechanisms [1, 2]. In *Trypanosoma brucei*, the level of an mRNA is determined by the number of gene copies, the decay rates in both nucleus and cytosol, and the processing efficiency [3]. The latter three processes are in turn strongly influenced by RNA-binding proteins [4–6].

*Trypanosoma brucei* is a digenetic extracellular parasite that replicates in the blood and tissue fluids of mammals (as bloodstream forms), and the digestive system of tsetse flies (procyclic forms are found in the midgut). ZC3H32 (Tb927.10.5250) is a 72 kDa protein with three non-canonical CCCH zinc finger domains [7]. By quantitative mass spectrometry, ZC3H32 is about 20 times more abundant in bloodstream forms than procyclic forms, with five phosphorylation sites near the C-terminus [8, 9]. This regulation is achieved on two levels: *ZC3H32* mRNA is 2–4 times more abundant, and stable, in bloodstream forms than in procyclic forms [10–12], and it is more efficiently translated in bloodstream forms [10]. Correspondingly, results from a high-throughput RNAi screen indicated that ZC3H32 is required for proliferation of bloodstream forms, but not procyclic forms [13]. CCCH zinc finger domains usually bind RNA, and indeed, ZC3H32 was detected in a mass spectrometry analysis of proteins that cross-link to polyadenylated RNA in bloodstream forms *in vivo* [14].

MKT1 is a protein that forms a complex with PBP1, LSM12 and poly(A) binding protein (PABP), both in yeast and in trypanosomes [15]. Extensive experiments with the components of the trypanosome complex demonstrated that attachment of any of its component proteins to the 3' untranslated region of a reporter mRNA caused an increase in the reporter mRNA abundance and translation; most likely, this happens because of stable attachment of PABP via the PBP1 subunit [15]. We originally identified trypanosome MKT1 as an interaction partner of ZC3H11, an RNA-binding protein that is required for stabilization of heat shock protein mRNAs [15]. In the course of that work, we identified ZC3H32 as an interaction partner of MKT1 in a yeast 2-hybrid screen, and two ZC3H32 peptides were identified by mass spectrometry of an affinity-purified MKT1 preparation [15]. We speculated that recruitment of the MKT1 complex by ZC3H32 should result in stablization of any mRNAs that are associated with ZC3H32. However, when ZC3H32 was artificially attached to a reporter in a "tethering" screen, results suggested instead that expression was suppressed [14].

The observations so far suggested that ZC3H32 is an mRNA-binding protein with a specific role in suppressing mRNA translation, and/or enhancing mRNA degradation in bloodstream-form trypanosomes. In this paper, we set out to test this idea. Although we could confirm the suppressive action of ZC3H32, we were unable to find any evidence for sequence specific RNA binding.

## Results

### Conservation of ZC3H32

ZC3H32 is a 655-residue protein with three zinc fingers. These vary slightly from the consensus C-x8-C-x5-C-x3-H structure: their arrangements (from N to C-terminus) are C-x7-C-x5-C-x3-H, C-x9-C-x5-C-x3-H, and C-x7-C-x4-C-x4-H. ZC3H32 homologues are present in all Trypanasomatida genomes sequenced to date, including all salivarian trypanosomes, *Leishmania*, *Endotrypanum*, and *Crithidia* and the more distantly-related *Angomonas* (an insect gut parasite with bacterial endosymbionts) and *Bodo saltans*, a free-living bacteriovore. The sequence alignment (S1A Fig) reveals a conserved core of about 200 residues containing the three CCCH domains, separated by predicted alpha-helices (S1C Fig). For residues 258–363 (containing the CCCH domains) all homologues showed at least 60% identity, and residues 199–373 were almost as well conserved. This suggests that the proteins may have similar RNA binding specificities. The N- and C-termini, in contrast, differ very considerably between species both in primary sequence and predicted secondary structure. The closest resemblances for the N- and C-termini are between *Leishmania*, *Endotrypanum* and *Crithidia*, with surprising differences even between the salivarian trypanosomes. Residues 138–145 of *T*. *brucei* ZC3H32 are KHTNNPY, which is related to the known MKT1 interaction motif (Y/W/T)(R/T/Q)H(N/D)PY [15]. In a two-hybrid screen with MKT1 as bait, all interacting fragments of ZC3H32 included this motif (S1B Fig) [15]. (K/R)HTNNPY is present in the *T*. *brucei*, *T*. *congolense*, and *T*. *cruzi* ZC3H32 sequences but not in *T*. *vivax*, another salivarian trypanosome. *Leishmania*, *Endotrypanum*, and *Crithidia* also lack it. In contrast, *Angomonas* has SYSNNPY and *Bodo* has KRNNPY (S1A Fig). This suggests that the motif was present in the common ancestor [16], but has been lost at least twice independently. Documented ZC3H32 phosphorylation sites [17] are not conserved at all (S1A Fig).

### ZC3H32 is mostly in the cytoplasm

In order to test the function of ZC3H32 with MKT1, we made bloodstream-form trypanosomes expressing three different tagged versions of the protein. To express approximately normal levels of ZC3H32 with a short tag, we integrated a sequence encoding the V5 tag into the genome such that the tag would be at the ZC3H32 N-terminus (V5-ZC3H32, S2B Fig) [18]. The protein was functional because the untagged allele could be knocked out (S2C Fig). In addition, we made cells in which ZC3H32 with two C-terminal myc tags was expressed under control of a tetracycline-inducible promoter (S2D Fig). When this tagged protein was expressed, both original gene copies could be deleted (S2E Fig). Finally, we made cells in which one copy of ZC3H32 had an N-terminal tandem affinity purification tag (TAP, protein A and calmodulin binding peptide), and the other had been replaced by a blasticidin resistance cassette (S2F Fig).

**Fig 1:**
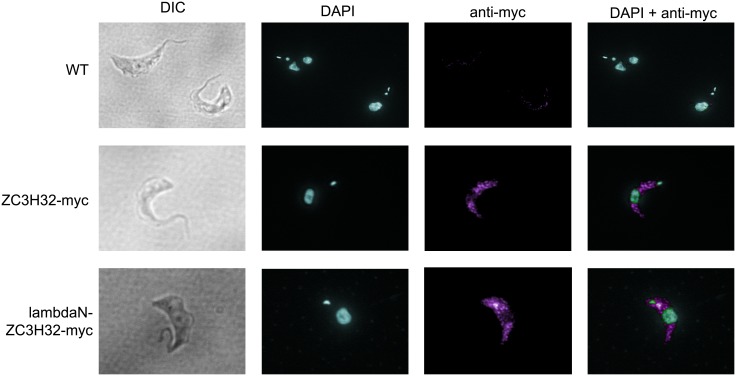
Myc-tagged ZC3H32 is in the cytoplasm. ZC3H32-myc was detected by anti-myc antibody and DNA (nuclei and kinetoplasts) were detected with diamino pimelic acid (DAPI). Differential interference contrast (DIC) images are on the left. The lowest panels show that lambdaN-ZC3H32-myc has a similar location.

If ZC3H32 were involved in mRNA control, as is implied by its *in vivo* cross-linking with mRNA, we would expect to find it in the cytosol. ZC3H32-myc was examined by immunofluorescence and it was indeed found in the cytosol (Fig 1). The distribution was slightly granular but this could have been a fixation artifact.

**Fig 2:**
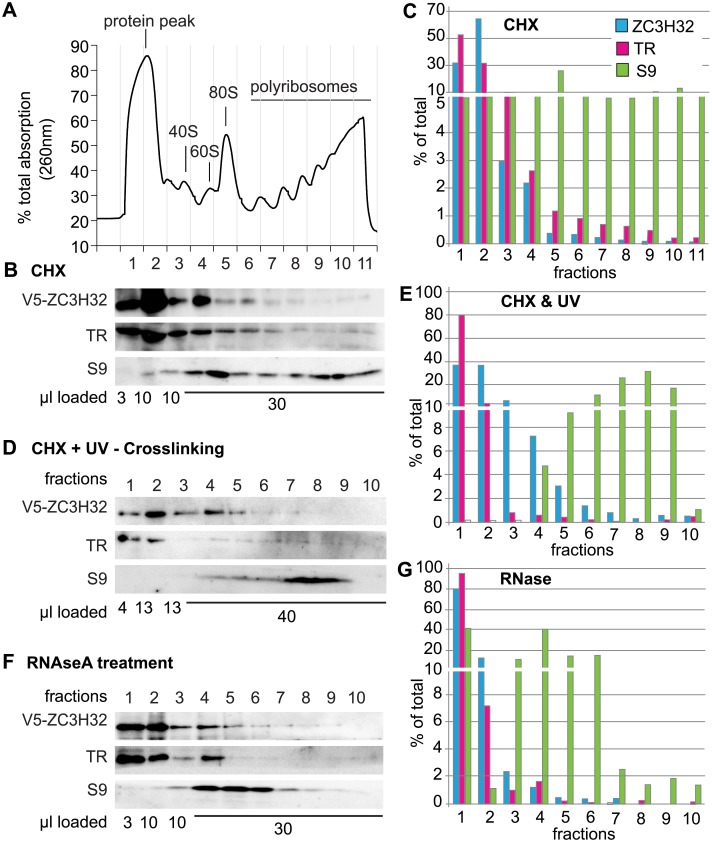
No evidence for association of ZC3H32 with polysomes. (A) A typical optical density profile after sucrose density gradient centrifugation of extracts from trypanosomes expressing V5-tagged ZC3H32, and treated briefly with cycloheximide. Fraction numbers are below; the heaviest fraction is fraction 11. (B) Typical Western blot showing the distribution of V5-ZC3H32 for (A), trypanothione reductase (TR) and ribosomal protein S9. (C) Quantitation of (B), with results adjusted for loading. Note the interruption of the scale. (D) As in (B), but the trypanosomes were UV-irradiated prior to lysis in order to bind proteins to RNA. 10 fractions were obtained. (E) Quantitation of (D)—as in C. Results are also similar to (C). (F) As (B) but 100 μg/ml RNase was included in the polysome buffer. (G) Quantitation of (F).

To find out whether ZC3H32 is associated with polysomes, we examined the distribution of V5-ZC3H32 on sucrose gradients (Fig 2A–2C). The distribution was not perceptibly different from that of trypanothione reductase, a cytosolic enzyme and was unaffected by either UV cross-linking (Fig 2D and 2E) or RNase treatment (Fig 2F and 2G). We concluded that only a very small proportion of ZC3H32 could be polysome associated. This suggests either that ZC3H32 is present in considerable molar excess to its mRNA targets, or that these targets are not being translated.

### ZC3H32 is essential in bloodstream form trypanosomes

**Fig 3:**
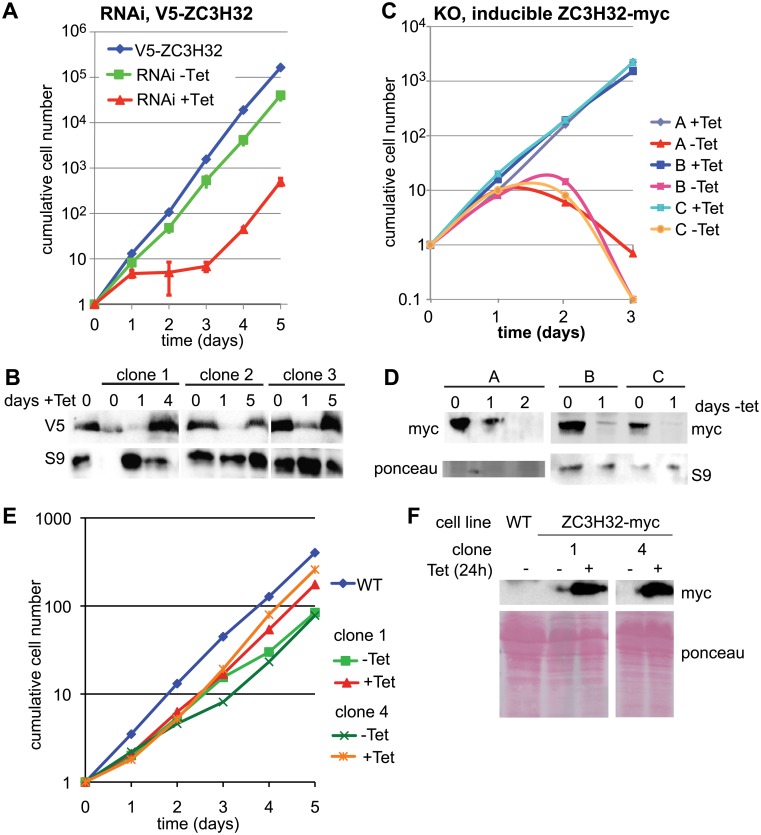
ZC3H32 is essential in bloodstream-form trypanosomes. (A) Results with a trypanosome clone expressing the tet-repressor and with a V5 tag sequence integrated into the genome in frame with ZC3H32 (S2B Fig). RNAi was induced by addition of tetracycline, in 3 clones, and cells were diluted as necessary. Cumulative cell numbers after various days of incubation (means and standard deviation) are shown. (B) Expression of V5-ZC3H32 after induction of RNAi—control for (A). Expression is decreased on day 1, but has recovered on days 4–5; cell growth is also restored (panel A). Antibody to ribosomal protein S9 serves as a loading control. (C) Cells containing an inducible copy of ZC3H32-myc, and no endogenous copies (S2E Fig) were grown with or without tetracycline. ZC3H32-myc expression was lost after withdrawal of tetracycline. Cumulative cell numbers after various days of incubation are shown for three clones. Cells were diluted as necessary. (D) Expression of ZC3H32-myc after withdrawal of tetracycline, experiment (C). (E) Growth of two cloned procyclic-form cell lines with tetracycline-inducible expression of ZC3H32-myc. (F) Western blots showing expression of the myc-tagged protein for (E).

To check the results of the published RNAi screen [13], we decreased expression of ZC3H32 and measured cell growth. Depletion of ZC3H32 by RNAi inhibited growth in four independent cloned lines, but the cells recovered protein expression and growth after 4–5 days (Fig 3A and 3B). In contrast, when we placed cells with only a single, tetracycline-inducible copy of ZC3H32-myc (S2E Fig) in medium without tetracycline, the cells died (Fig 3C and 3D). After 15 or 24h of tetracycline withdrawal, ZC3H32-myc was undetectable (S3A Fig) but there was no major effect on total protein synthesis (S3C Fig), and at 15h, also no clear effects the numbers of cells in different cell cycle stages (S3D Fig). Our attempts at RNAi in procyclic forms were unsuccessful: little decrease in the transcript level was found even though six different lines were tested. (Protein was not measured.) Without doing a knockout, we could therefore neither confirm, nor contradict, the previous result suggesting that ZC3H32 is not required in procyclic forms [13]. Inappropriate expression of ZC3H32-myc in procyclic forms, however, did not impair growth (Fig 3E and 3F).

### ZC3H32 association with MKT1

**Fig 4:**
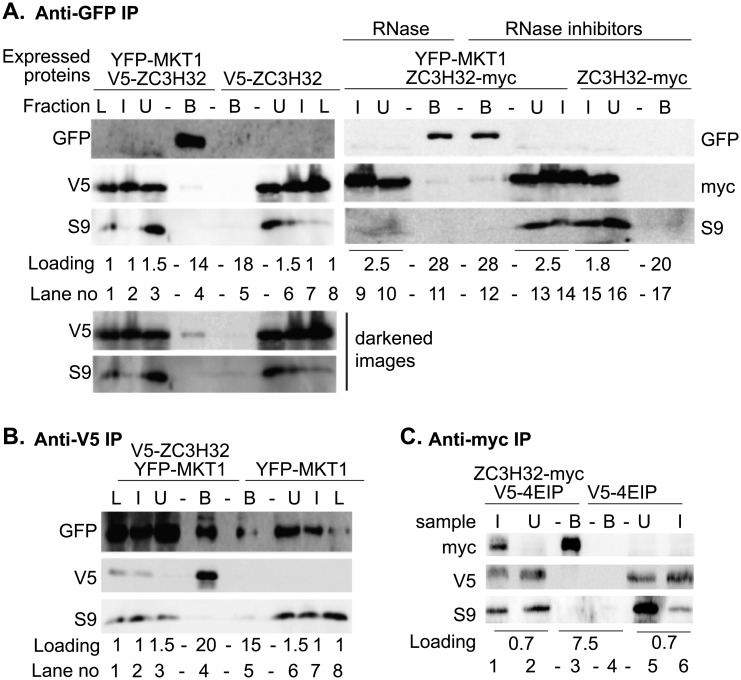
Most ZC3H32 is not associated with MKT1. (A) Trypanosomes expressing V5-ZC3H32 from the endogenous locus, or with induced (RNA- polymerase-1-mediated) expression of ZC3H32-myc, with or without YFP-tagged MKT1. Extracts were immunoprecipitated with anti-GFP and proteins detected by Western blotting. The loading (in cell-equivalents relative to the input) is indicted below the blots. An artificially darkened image (increased exposure but no contrast adjustment) is also shown for V5-ZC3H32. (B) As (A), but with Immunoprecipitation of V5-ZC3H32 and detection of YFP-MKT1. (C) Anti-myc immunoprecipitation of extracts from cells expressing V5-4EIP, with or without ZC3H32-myc.

ZC3H32 clearly interacted with MKT1 in a two-hybrid screen, which was done with full-length MKT1 as bait, and protein fragments as prey [15]; and fragments might be mis-folded. ZC3H32 also was found after tandem affinity purification of MKT1, but the purification was done only once, ZC3H32 coverage was just 5%, and a pull-down via RNA could not be excluded [15]. Thus it was not at all certain that full-length ZC3H32 interacts with MKT1 inside trypanosomes. To test this more rigorously, we expressed V5-ZC3H32 or ZC3H32-myc in cells in which one of the *MKT1* open reading frames was N-terminally tagged with *YFP* [15, 19]. Very small proportions—less than 1%—of both tagged versions of ZC3H32 were pulled down with YFP-MKT1; the pull-down was not RNase sensitive and depended on the presence of YFP-MKT1 (Fig 4A). The anti-V5 immunoprecipitation also reproducibly pulled down YFP-MKT1 but the result was the same if no V5-tagged protein was present (Fig 4B). The results thus suggested that a very minor portion of ZC3H32 might be associated with MKT1 in bloodstream-form trypanosomes.

Our subsequent results (see below) suggest that full-length ZC3H32 can suppress expression of bound mRNAs. The strongest suppressor in the tethering screen was the putative translation regulator 4E-IP [14]. We therefore investigated whether ZC3H32-myc interacted with *in situ* N-terminally V5-tagged 4E-IP. The results were, however, negative (Fig 4C).

### Tethered ZC3H32 decreases reporter mRNA translation and abundance

**Fig 5:**
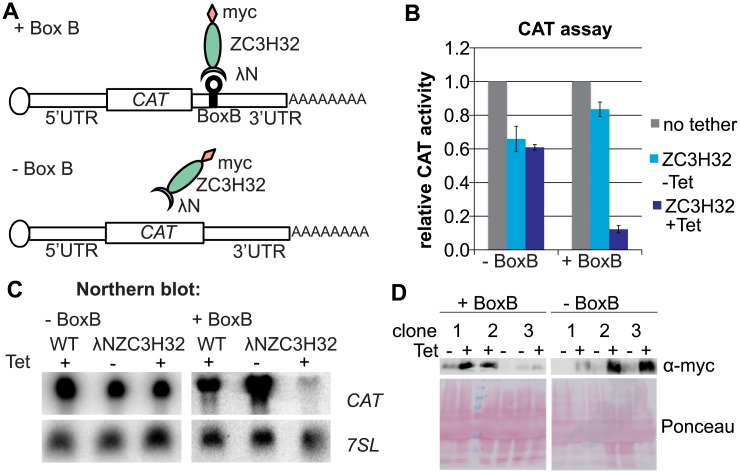
Tethering of ZC3H32 to a reporter mRNA represses expression. (A) Principle of the tethering assay. The reporter mRNA has a chloramphenicol acetyltransferase (*CAT*) open reading frame. The capped spliced leader is shown as a circle. Five boxB sequences (shown as a single lollipop) bind to the lambdaN peptide. In our case the lambdaN peptide is at the N-terminus of a ZC3H32 open reading frame with a C-terminal myc tag. The negative control reporter (lower diagram) has no boxB. (B) Effect on CAT enzyme expression from tethering lambdaN-ZC3H32-myc to the *CAT* reporter mRNA. CAT activity is shown as a proportion of that seen in cells with no tethered protein. Results are arithmetic mean ± standard deviation for three clones. (C) Effect on *CAT* mRNA of tethering lambdaN-ZC3H32-myc in the 3'-UTR. A typical Northern blot is shown. (D) Expression of lambdaN-ZC3H32-myc in the six different clones.

To test whether ZC3H32 can affect expression of bound mRNAs, we used a reporter RNA with a chloramphenicol acetyltransferase (*CAT*) open reading frame, and a truncated version of the actin (*ACT*) 3'-UTR. Between the open reading frame and 3'-UTR are five copies of a "boxB" sequence, which binds to the lambdaN peptide with high affinity. Expression of ZC3H32 with an N-terminal lambdaN peptide should result in binding of the fusion protein to the reporter (Fig 5A). LambdaN-ZC3H32 was in the cytosol (Fig 1). It strongly decreased CAT expression both at the level of protein (Fig 5B) and RNA (Fig 5C), whereas expression from a control reporter without boxB was unaffected.

**Fig 6:**
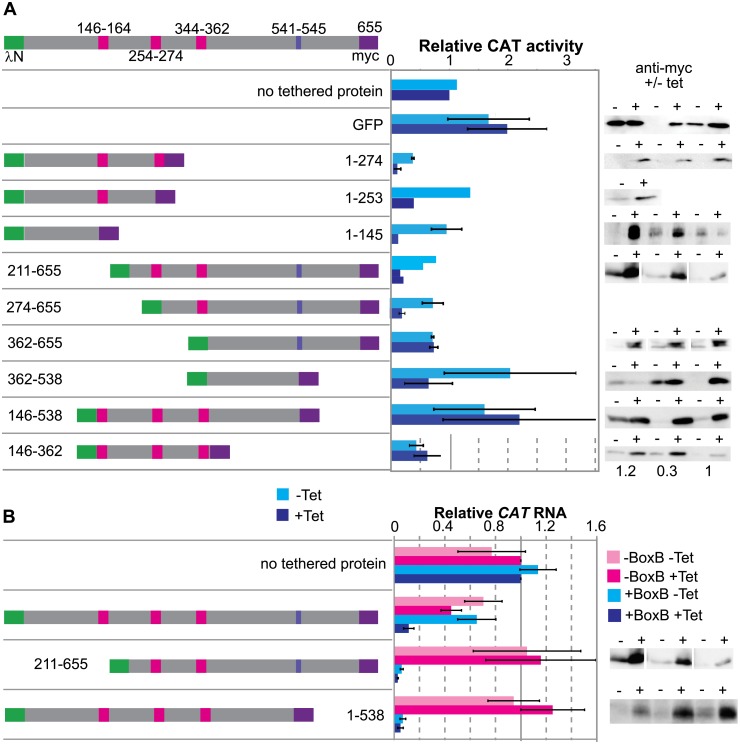
Both the N- and C-terminal portions of ZC3H32 repress expression in the tethering assay. A map of the complete tagged lambdaN-ZC3H32-myc protein is at the top, with the Cx_7_Cx_5_Cx_3_H zinc fingers in red, the Cx_9_Cx_5_Cx_3_H zinc finger in orange, and the putative MKT1 binding motif (NNPY from 541–545) in blue. Below, maps of expressed proteins are on the left and the effect on the reporter are on the right; cyan is the cell line without tetracycline and dark blue, the line with tetracycline. Western blots showing expression of the myc-tagged proteins are on the right. Only the anti-myc panels are shown; loading is shown in S4 Fig. Sometimes, there was easily detectable expression in the absence of tetracycline. (A) shows CAT activities relative to the amount with no tethered protein at all (top bars). Results are mean and standard deviations for 3 independent clones except for fragments 1–253 and 211–655, where only 1 and two clones, respectively, were measured. (B) shows relative RNA amounts, measured by quantitative Northern blotting using rRNA or *7SL* RNA as standards. Note that the Western blot used for construct 211–655 is identical to that in panel (A), since this was the same experiment.

We next investigated which parts of ZC3H32 were required for its action in the tethering assay by deletion analysis. We made cell lines with tetracycline-inducible expression, but often, the repression without tetracycline was incomplete, resulting in "leaky" control (Fig 6, S4 Fig). We induced expression for 24h then measured CAT activity. The results (Fig 6A) suggested that at least two regions of ZC3H32 could independently decrease expression. Deletion from the C-terminus showed that the N-terminal 145 residues—without the CCCH domains—were able to reduce CAT activity 10-fold. Lambda-N tagged fragments lacking this region (211–655 or 274–655) were also active. Further deletion from the N-terminus (362–655) seemed to reduce the repression (although perhaps expression of fully folded protein could have been lower), but a protein comprising all three zinc finger domains (146–538) was inactive. Overall the results suggested the presence of a second repressing domain towards the C-terminus. Unfortunately, expression of residues 529–655 ((K/R)HTNNPY to the C-terminus) was not detectable so the independent activity of this fragment could not be verified.

**Fig 7:**
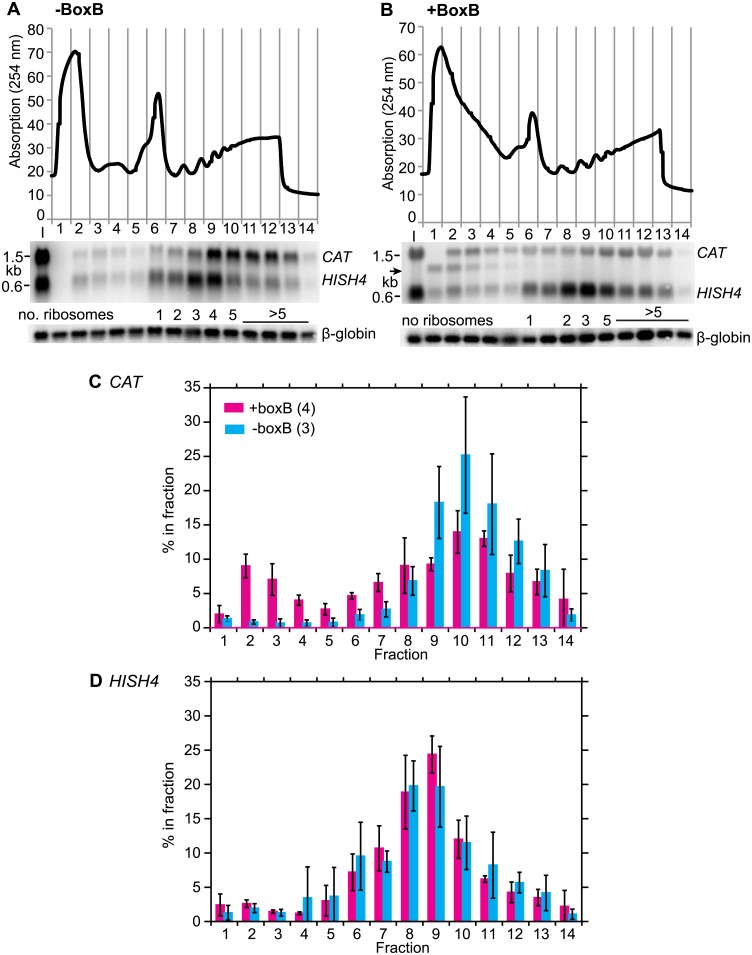
Tethering of ZC3H32 represses translation initiation. (A) Polysome profile of cells expressing the *CAT* reporter from Fig 5A without box B, and with expression of lambdaN-ZC3H32-myc. The top panel is the optical density reading across the gradient. Fraction 1 has the lowest sucrose concentration and fraction 14 the highest. A synthetic ß-globin RNA was added to the fractions before RNA preparation, as a control for the RNA purification efficiency. Below are the results from a typical Northern blot for the gradient fractions, hybridised with probes for *CAT*, histone H4 (*HISH4*) and ß-globin. (B) As (A), but with *CAT* reporter from Fig 5A with box B. (C) Percent of *CAT* RNA in each fraction, relative to the total signal of the whole gradient. The signals on the Northerns were measured from the phosphorimager files. Numbers of experiments are indicated and results are arithmetic mean ± standard deviation. (D) As (C) but for the control *HISH4* mRNA.

Next we examined the mechanism of repression in more detail. Tethering of full-length protein strongly decreased the amount of *CAT* mRNA (Fig 6B). Versions lacking either the N-terminal 210 residues, or the C-terminal 119 residues (including NNPY) did the same (Fig 6B; note that expression of both was present in the abscence of induction). After 24h induction of LambdaN-ZC3H32-myc expression, the *CAT-boxB* mRNA was barely detectable. To find out whether LambdaN-ZC3H32-myc tethering could prevent association of the reporter mRNA with polysomes, we therefore needed to induce expression for a shorter time. Fig 7 shows what happened when we induced for just 4h, which was sufficient to obtain a detectable LambdaN-ZC3H32-myc signal by Western blotting. The control *CAT* mRNA, without boxB, was concentrated mainly in the polysomal fraction, as expected (Fig 7A and 7C). In contrast, the *CAT-boxB* mRNA was found partially in the free fraction (Fig 7B and 7D). The distribution of a control mRNA, encoding histone H4, was not affected by expression of LambdaN-ZC3H32-myc. Tethering of ZC3H32 also caused the appearance of an additional *CAT* RNA, approximately 600 nt shorter, at the top of the gradient (Fig 7B, arrow). This could be explained by a cleavage just upstream of the boxB-3'-UTR segment. We have so far not seen similar fragments after tethering of any other protein to the same reporter, but this could be a technical issue, since our induction time was extremely short, the fragment had very low abundance relative to the total *CAT* RNA, and it was detectable only after gradient centrifugation.

### ZC3H32 expression, the transcriptome, and RNA binding

**Fig 8:**
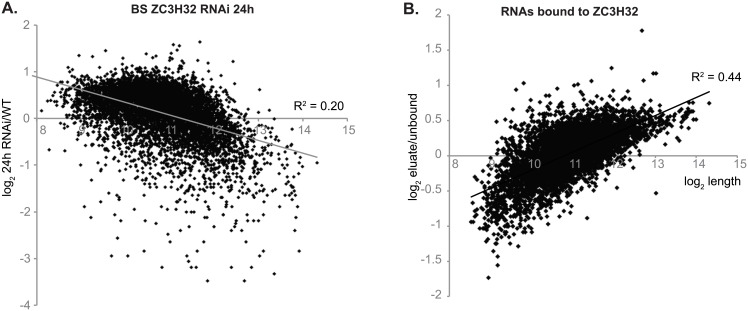
Transcriptome results. (A) *ZC3H32* RNAi was induced for 24h and the transcriptome (single experiment) was compared with two wild-type control transcriptomes using DESeq2. The result for each coding region (RNAi/Wild-type, log_2_-transformed) is on the y-axis and log_2_-transformed mRNA lengths (as annotated in TritrypDB) on the x-axis. The regression line (plotted using Microsoft Excel) and correlation coefficient are also shown. (B) Tandem-affinity tagged ZC3H32 was purified on an IgG column. RNA was purified from the bound and unbound fractions and analysed by RNASeq. The bound/unbound ratio was calculated based on the normalized read counts (reads per million reads). The graph is as in (A), except that shows the log_2_-transformed average ratio for triplicate experiments is on the y-axis.

To assess the effects of changes in ZC3H32 on mRNAs, we initially examined transcriptomes 24h after induction of RNAi in bloodstream forms (S3A Fig), or ZC3H32-myc expression in procyclic forms. This was done with single samples. If ZC3H32 is a repressor, we would expect its targets to increase after RNAi, and to decrease after expression of ZC3H32 in procyclic forms. After removal of genes with very low read counts, no mRNAs had this pattern (S1 Table, sheet 1). The changes after 24h RNAi were biased towards longer mRNAs (Fig 8). Moreover, mRNAs encoding numerous other RNA binding proteins and two components of the degradation machinery were strongly reduced, which made it impossible to tell which effects were specifically due to loss of ZC3H32. We independently checked expression of some procyclic-specific mRNAs which initially appeared to be regulated, but detected no clear effects (S5 Fig).

In an attempt to find direct effects of ZC3H32 loss before any growth inhibition, we used bloodstream forms containing a single tetracycline-inducible copy of ZC3H32-myc. Growth of this cell line is already slowing within 24h of tetracycline removal (Fig 3C), so we decided to measure effects just 12h after tetracycline removal. At this point, by Western blotting a trace of ZC3H32-myc was visible, suggesting that the protein had been reduced by 80% relative to the induced level (S3B Fig). Since we do not have an antibody to ZC3H32 itself, we do not know whether this level of ZC3H32 was lower than in wild-type cells. To find out whether ZC3H32 depletion influences mRNA translation or mRNA levels, we measured not only total RNA, but also polysomal and non-polysomal RNA. After 12h depletion the level of ZC3H32 mRNA was reduced by 95% relative to wild-type (S1 Table Sheet 2), and the fraction of ZC3H32 mRNA in polysomes also appeared reduced (S1 Table Sheet 3) although the results were not quantitatively reliable because the read counts were very low. Disappointingly, however, only one mRNA was significantly increased in total mRNA, and no RNA except *ZC3H32* was decreased. 123 mRNAs were significantly decreased in polysomal RNA after *ZC3H32* depletion, but most of these had such low read counts that the effects were probably random scatter. Loss of ZC3H32 should cause an increase in polysomal RNA for the targets, but only 24 mRNAs had this behaviour, and most of these were increased in the free fraction as well, despite showing no increase in total RNA. 37 mRNAs appeared to have shifted at least slightly towards the polysomal fraction, (S1 Table, sheet 3, columns Z and AA) but given the size of the dataset this would be expected from random variation. Overall the changes in these small numbers of mRNAs suggested only technical variations. We concluded that after 12h of ZC3H32 shut-off, there were no significant effects on either the transcriptome or polysomal loading.

The results so far suggested that transcriptomes should be analysed at several time points between 12 and 24h in order to detect specific effects. However first, it seemed sensible to try to find out whether ZC3H32 was even associated with a specific subset of mRNAs. For this, we used the bloodstream-form cells expressing only ZC3H32 bearing an N-terminal TAP tag. After purification on IgG columns, we compared the bound and unbound RNAs from three replicates. The *ZC3H32* coding region itself was clearly enriched: this can happen when the mRNA is pulled down via the TAP-tagged nascent polypeptide (S2 Table). However, only one other mRNA was reproducibly even 2-fold enriched. Moreover, there was a clear correlation between column binding and mRNA length (Fig 8B). This could mean that ZC3H32 binds to mRNAs without sequence specificity, but it could also just reflect non-specific binding or trapping of mRNAs, or their bound proteins, by the resin. Preliminary attempts to detect binding using an iCLIP protocol also failed. Since these results provided no evidence for sequence-specific RNA binding by ZC3H32, we decided not to repeat the depletion experiment.

### ZC3H32 association with other proteins

Finally, we resumed attempts to identify a mechanism by which tethered ZC3H32 might cause translation suppression or mRNA decay, by looking for protein interaction partners. Tandem affinity purification of TAP-ZC3H32, followed by mass spectrometry, identified 10 proteins that were at least 3-fold more abundant than in a GFP-TAP control in three experiments (two without RNase, and one with RNase) (S3 Table). None of the partners was obviously linked to mRNA decay but fragments of two—the SMN protein and an enzyme (adenylosuccinate synthetase)—repressed expression in the tethering screen [20]. In procyclic trypanosomes, SMN is detected only in the nucleus, where it is—as in other eukaryotic cells—required for snRNP assembly [21]. Since ZC3H32 is in the cytosol and only present in bloodstream forms, it is extremely unlikely that ZC3H32 has a role in snRNP assembly. It is, however, possible that SMN has an additional, cytosolic, function in bloodstream forms in which ZC3H32 is involved. Interestingly, all of the potential interacting proteins except SMN had been identified in several other mRNP-related purifications, suggesting that they are indeed associated with aspects of mRNA metabolism. This was also true of some additional proteins that were associated in an RNA-dependent fashion, or had lower coverage. Perhaps the most interesting of the latter group was the NEK9 protein kinase, which also co-purified with, and had a two-hybrid interaction with, MKT1 [15].

## Discussion

ZC3H32 expression is bloodstream-form specific, it cross-links to mRNAs in vivo, and its loss is very rapidly lethal. We therefore expected that ZC3H32 would have a specific function in suppressing expression of particular genes in bloodstream-form trypanosomes. Although this may indeed be the case, we were unable to detect such a function with the methods employed. We indeed found that ZC3H32 is in the cytosol, and that it reduced translation and caused mRNA degradation when attached to an mRNA. Investigation of proteins that interacted with ZC3H32, however, did not yielded any clue as to its mode of action. After tandem affinity purification, the nine proteins that were found to be associated specifically with ZC3H32 (and not with similarly purified TAP-GFP) were (with one exception, SMN) also found after purification of a variety of other mRNP proteins, such as ZC3H11 and MKT1 [15] or DRBD18 [22]. None of these proteins suppresses expression after tethering [20], and none binds to mRNA [14]. Three of them are not even in the cytosol, suggesting that the association might have occurred after cell lysis.

The presence of ZC3H32 in Euglenids that are either monogenetic parasites, or free-living, and the lack developmental regulation of *ZC3H32* mRNA levels in *Leishmania* [23] or *T*. *cruzi* [24] argue against a conserved role in developmental regulation. Since only the RNA-binding domain is well conserved, it is possible that ZC3H32 has species-specific functions. However our results for *T*. *brucei* also provided no convincing evidence for developmental regulation of specific mRNAs. Most importantly, we were unable to demonstrate any sequence-specific mRNA association. One possible reason for this result could be that the targets have extremely low abundance; however we have recently succeeded in identifying targets of another repressing protein, RBP10, using exactly the same procedure (E. Mugo and C. Clayton, manuscript submitted and [25]). Another argument against a role for ZC3H32 in suppressing expression of specific, procyclic-specific mRNAs is that expression of ZC3H32 in procyclic forms did not affect trypanosome proliferation—although target mRNAs might be protected from ZC3H32 by competing procyclic-specific RNA-binding proteins. Finally, depletion of *ZC3H32* for 24h resulted only in preferential loss of longer mRNAs. This latter type of transcriptome pattern has been seen in trypanosomes with growth-inhibitory depletion of several other proteins implicated in cytosolic mRNA metabolism: PUF2, MKT1, PAN2 and CAF1 [26]. Moreover, the same effect is seen even more strongly upon loss of CTR9 [27], a polymerase II transcription elongation factor. The selective loss of longer mRNAs could therefore be a side-effect of a reduction in the rate of transcription elongation [26].

Overall, therefore, it seems possible that ZC3H32 has a general role in controlling overall rates of mRNA decay or translation in bloodstream forms. If so, perhaps ZC3H32 function is taken over by a different protein in procyclic forms.

## Materials and methods

### DNA manipulation and trypanosomes

PCR reactions were done using GoTaq (Promega) polymerase or, for long open reading frames, Q5 Polymerase (New England Biolabs). Trypanosome Genomic DNA was isolated using the illustra GenomicPrep cells and tissue DNA isolation kit (GE Healthcare). Bloodstream-form trypanosomes were grown in HMI-9 (10% serum), at densities between 5x10^4^/ml and 2x10^6^/ml; procyclic forms were grown in MEM-Pros (10% serum) between 5x10^5^/ml and 5x10^6^/ml. Tetracycline-regulated transcription was induced with of 0.1μg/ml tetracycline. Bloodstream cells were usually harvested at densities of 5x10^5^ - 1x10^6^ cells/ml, procyclic forms at 1–5 x10^6^ cells/ml. Plasmids and oligonucleotides used in this study are listed in S4 Table, and some of the generated cell lines are shown in S1 Fig.

### Protein analysis

For Western blotting, 0.2–1.0x10^7^ cells were pelleted, resuspended in 2xLaemmli buffer and boiled for 5min at 95°C. The proteins were transferred to nitrocellulose, stained with Ponçeau red, blocked for at least 1h with 5% skimmed milk powder in TBS-T (50 mM Tris; 150 mM NaCl; 0.05% Tween 20) with gentle agitation at room temperature (except incubations over night). The blot was incubated with the primary antibody for at least 1 hour, using antibodies dissolved in 5% defatted milk. Blots were washed 3 times for 5 min with 1xTBS-T, then for at least an hour with a horseradish-peroxidase conjugated secondary antibody. Before detection, the blot was washed three times for 10 min with 1xTBS-T. Bands were detected using the Western lightning Plus or Ultra ECL^T^ detection reagents (Perkin Elmer). If necessary, the antibodies were removed using stripping buffer (25mM glycine; 1%SDS, adjusted to pH2 with HCl) and new antibodies were applied. Immunofluorescence was performed on formalin-fixed, detergent-permeabilized parasites [28, 29], using anti-myc, anti-V5 and PAP antibodies from Abgene (mouse, 1:500).

For TCA precipitation, 1/5 volume of 100% TCA was added, the mixture was vortexed, then it was incubated at -20°C overnight. The precipitate was sedimented for 15min at room temperature in the microfuge, and washed with 3 volumes of the original sample volume of acetone, with 10min incubation at room temperature each time. The pellet was dried for 10min and then either stored at -20°C or dissolved in 1x Laemmli buffer.

Chloramphenicol acetyltransferase was assayed as previously described [30].

Total protein synthesis was assessed by labeling with [^35^S]-methionine as described in [31].

### Immunoprecipitation

1–2^.^10^8^ cells were sedimented at 2300g at 4°C for 10min, washed twice with ice-cold PBS, then snap-frozen and stored at -80°C. The cell pellet was thawed on ice and dissolved in 300μl lysis buffer (10mM Tris-Cl; 10mM NaCl; 0.2% IGEPAL; 2mM DTT; pH = 7.4; complete protease inhibitor (Roche)). If needed, 0.5μg RNAseA or 6μl RNAseIN (RNase Inhibitor, promega) were included. Cells were broken by passing the them approximately 50 times through a 21 gauge needle. The lysate was spun at 13.000g for 5min at 4°C to remove the cell debris. Before immunoprecipitation the NaCl concentration of the cleared lysate was increased to 100-150mM.

30–50μl α-V5, α-myc (both from Abgene) or GBP (Green fluorescent protein binding) beads were washed three times with IP buffer. The cleared lysate was added to the beads and incubated for 2h on a rotary shaker at 4°C. The beads were washed three to five times with IP buffer then boiled in Laemmli loading buffer.

### RNA preparation and Northern blotting

RNA was usually isolated from 2-10x10^7^ cells. The cells were pelleted by centrifugation, resuspended in 1ml pegGOLD TriFast^™^ (Peqlab, GmbH), and incubated for 5 min at room temperature. After chloroform extraction the RNA was precipitated with isopropyl alcohol, washed with 75% ethanol, air-dried for 5-10min and dissolved in sterile water.

For Northern blotting, approximately 10μg RNA was separated by formaldehyde gel electrophoresis, blotted to a Nytran N membrane, UV cross-linked, and RNA visualised using methylene blue. The ^32^P-dCTP-labeled probes were made using the Prime-It^®^ RmT Random Primer Labeling Kit (Stratagene), purified using the Qiagen nucleotide removal kit, then hybridised with the blots. The signal was detected by phosphorimaging and quantified using ImageJ.

### Polysome analysis

Cyclohexamide (final 25 μg/ml) was added to cultures of 3-4x10^8^ cells, incubated at room temperature for 1 min, then cells were chilled in a dry ice ethanol bath. They were sedimented, then either lysed immediately resuspended in freezing buffer (1x Polyribosome buffer, 200mM sucrose, 10% glycerol, 100 μg/ml cycloheximide) and kept at -70°C. Frozen pellets were thawed on ice and washed twice (4°C, 2min, 3000rpm) with wash buffer.

The cells were then resuspended in Polyribosome buffer (10mM Tris pH 7.4, 10mM MgCl_2_, 300mM KCl) with 200mM sucrose, Roche complete protease inhibitor without EDTA, 2mM/ml DTT 0.4mg/ml Heparin, 10μg/ml Leupeptin and 100μg/ml Cycloheximide and 0.2% NP-40, and lysed by 50 passes through a syringe needle). The lysate was cleared by centrifugation (4°C, 4,500rpm, 5min, microfuge) and loaded onto 15%-50% sucrose gradients in polysome buffer with 400μg/ml heparin, 2mM DTT and 10μg/ml leupeptin. Gradients were spun at 4°C, either at 36000rpm for 2.5h or at 40,000rpm for 2h. For 14x89mm tubes the SW41 rotor, for 11x60mm tubes the SW60 rotor was used. The fractions were collected and the OD_260_ profile measured using an ISCO 160 gradient former. For Western blotting, 30–50μl of fractions 4 onwards were loaded on the gel; for the first three fractions, 5–15 times less was loaded. For RNA isolation, the samples were mixed with 3 parts peqGOLD TriFastFL and processed according to the manufacturer’s description.

### Affinity purification, mass spectrometry and RNASeq

To study interactions of ZC3H32 we used bloodstream-form trypanosomes in which one *ZC3H32* allele was knocked out and the other bore a sequence encoding an N-terminal TAP tag. For protein partners, TAP-ZC3H32 was purified on an IgG column, bound protein was eluted with Tobacco Etch Virus (TEV) protease, bound to a calmodulin column and eluted with EGTA [32, 33]. Proteins were subjected to denaturing SDS-PAGE and analysed by mass spectrometry. For RNA binding, only the first purification step was applied. The bound and unbound RNAs were purified. For RNASeq, RNA preparations were rRNA-depleted using rRNA-complementary oligonucleotides and RNase H [34], except with the 24h RNAi experiment which was done using poly(A)-selected RNA. Library-building and sequencing were done using standard Illumina protocols (see E-MTAB-5612 and E-MTAB-4558). The reads were aligned using Bowtie2, allowing for up to 20 alignments and one mis-match, then all reads aligning to open reading frames were counted using custom scripts [34]. The data were analysed using DeSeq2 [35] Automated scripts are available for both the sequence alignments [36] and DeSeq2 analysis, including functional class enrichment [37].

## Supporting information

S1 Fig Protein sequence analysis. (A) Sequence alignment of ZC3H32 from *T*. *brucei* TREU927 (Tb) (XP_822770.1), *T*. *congolense* (Tco) (CCC93676.1), *T*. *cruzi* (Tc) (EKF99112.1), *T*. *vivax* (Tv) (CCC51478.1), *Leishmania major* (Lmj) (XP_001686656.1), *Endotrypanum monterogeii* (Emt), *Crithidia fasciculata* (Cf) (CFAC1_250016000 in TritrypDB), *Angomonas deanei* (Ang) (EPY19775.1) and *Bodo saltans* (Bs) (CUF98976.1). The Cx_7_Cx_5_Cx_3_H zinc fingers are indicated with magenta and cyan shading, the Cx_9_Cx_5_Cx_3_H zinc finger is indicated with orange and cyan shading, and the putative MKT1 binding motif (NNPY) with purple shading. Mapped phosphorylation sites in the *T*. *brucei* protein [17] are indicated with a red "P". (B) Interactions with MKT1—Mapping of reads in the yeast two-hybrid screen [15]. The blue dots show reads after screening with the empty vector; the red ones are from using MKT as bait. The position of each dot shows the beginning of the peptide encoded by the interacting clone. The NNPY sequence of ZC3H32 starts at position 541, and only sequences that started upstream of this were able to interact with MKT1. (C) ZC3H32 protein secondary structures predicted using DNAStar. The pink segments at the top are the CCCH domains and the blue block is the NNPY.
(PDF)

S2 Fig Cell line characterization. (A) Schematic structure of the *ZC3H32* gene. Oligonucleotides used for amplifications are indicated. (B—F) construction of different cell lines, with PCR verification results. WT: wild-type; V: one V5-tagged copy, one normal copy; m: myc-tagged copy without knock-out. BSD: blasticidin S deaminase gene, PAC: puromycin resistance gene.
(PDF)

S3 Fig ZC3H32 depletion for up to 24h has no effect on overall protein synthesis and does not cause specific cell cycle arrest. All experiments were done in cells with a single copy of tetracycline-inducible ZC3H32-myc (inducible knock-out or iKO). (A, B) Levels of ZC3H32-myc after tetracycline withdrawal. Cells were centrifuged, as much medium as possible was removed, and then cells were resuspended in tetracycline-free medium, which should reduce the tetracycline concentration to below 5 ng/ml. In panel B we also checked the effect of washing with PBS but this was clearly not necessary. The lower panel is the Ponceau-red stained membrane. (C) Cells were pulsed with [^35^S]-methionine at various times after tetracycline withdrawal. The Coomassie-stained SDS-PAGE is on the left and the autoradiogram is on the right. For quantification the overall density on each lane of the autoradiogram was normalised to that of the Coomassie stain. The results for experimental lanes were expressed relative to the average for the two wild-type controls, which was set at 100%. (D) Cell cycle stages of trypanosomes after tetracycline withdrawal. The numbers of nuclei (N) and kinetoplasts (K) were counted. 1N1K cells are in G1 phase; 1N-bigK are replicating kinetoplast DNA and will be in S phase; 1N2K is late S or G2; and 2N2K is after mitosis but before cytokinesis.
(PDF)

S4 Fig Expression of different fragments of lambdaN-myc tagged ZC3H32; controls for Fig 6. (A) Fragments; the Ponceau stain or ribosomal protein S9 are shown as a controls. (B) Full-length protein.
(PDF)

S5 Fig Effect of ZC3H32 depletion on amounts of selected developmentally-regulated mRNAs in bloodstream forms. (A) Western blot after tetracycline removal, inducible knock-out cell line. The remaining blots are Northern blots, with quantitation using the *7SL* RNA as a loading control. (B) Glycosomal malate dehydrogenase, gMDH. (C) Repeat of gMDH, plus succinyl coA synthetase (alpha subunit); the results from two RNAi experiments are also indicated. (D) Repeat for succinyl coA synthetase. (E) Isocitrate dehydrogenase.
(PDF)

S1 Table Transcriptomes of trypanosomes after ZC3H32 depletion. For detailed legend see sheet 0.
(XLSX)

S2 Table RNAs associated with affinity-purified ZC3H32. For detailed legend see sheet 0.
(XLSX)

S3 Table Proteins associated with tandem-affinity-purified ZC3H32. For detailed legend see sheet 0.
(XLS)

S4 Table Plasmids and primers. (DOCX)
