# Supplementary material for: The role of the zinc finger protein ZC3H32 in bloodstream-form Trypanosoma brucei
Source: PLoS One. 2017 May 17;12(5):e0177901. doi: 10.1371/journal.pone.0177901 (PMC5435347; doi:10.1371/journal.pone.0177901)

A. Alignment

```
Tb9  -MSGTNHLRQVVPAAVY-NPIPPAPPFMVFCDPY---------AKVANATAKYRNQPPVAPNAA-------------AVF 56
Tco  -MSGSGNPRQGHPVAGYRDPLSSAQSVMMFCGTN---------MSSSGVPISFRSHSHVTANPA-------------TVF 57
Tv   -MLGNIPLQKSFHSPGVPNPRAPLQTLMMMYDVY---------PKTSTVATGFANTAAVAAVP--------------AAF 56
TcM  -MFGSEQFRQRFPVGGGSNPAAAATPFMMMYGVC---------PKTNCVASNYSNQPQVTTNN--------------AAF 56
Lmj  ------MIYKAYGSP--ENNSGNTHMYSMYAPPN------AYVQGQGGSRTMYGYAGTGGHGSGHP--FGYHANPSMGVT 64
Emt  MYGDQKMMYNVYGTL--AHSGNNTQMFSTYAPLN------AYGSGHGGGSMMYDYAVTGGQGTGQP--YGYHTDPSVGAT 70
Cf   MYHGSDGMYNVYSGHGNTNSGAGNGQYSMYNNHNNGGGMYGNVPGMHNSPMMYVYGNAGNSSPSAAGGYGMRVDPAVAAS 80
Ang  MMDNTFQQTSYYFDP----SSMNNGNGNFYVSNN--------NPGMNGIPMSNVNQQNGAFGT-----FAFSPNNNTNAL 63
Bs   MYYGGHEDLGTFVVQSSTNQQQQQQYGRNNGGGGRGGRRGGGGGGPAEFSAARNVQVMPNPTAYIAGG-QMNWGTPMT 79

Tb9  QQQLYSLPFHGVAGVPAPMREE----------------------------------EDADGSFEVHDSCLKAPITVGRRVV 103
Tco  QPAVYGYPLPGVGSLPAPIRED----------------------------------DDADTPFEAHDNHLKAPITVRRNVV 104
Tv   THRLYGTPVSALTGLPSAAFD-----------------------------------EDAGGSFEVHDSHLKAPIVVQRSVV 102
TcM  -QRPYGFPVNGYGATSTAPHEE----------------------------------EDTEGLFEVHDSHWKVPISVHSSVV 102
Lmj  YEYNGGINVHHNGSAYRVG----GGTN------GRDRGGDVVSLRN----LHGYDQQATSDYEVHDNQLKAPVVVTPATV 130
Emt  YEYTGGNNVHSVGGVYRVYGARVGGTN------GCARVGDTNGARN----LQGYDQPATFDYEVHDNQLKAPVVVTPATV 140
Cf   YEYNG---INHIGGGRGRGGRGAGGYNTSPMHPAHARRGGSNFFRGGGGGGNGYDQQAPSDYEVHDNQLKAPVAVTMETV 157
Ang  MASNNNISTNSVGNTSVMSSNNPNLNNNNNNMGFPMQMGMMQMNQVWTPIVSGNNTAFQGEFEVHDSQLKAPITIRPTDV 143
Bs   FTTQQQQLPHHQQQQQQQHHFSPSRGNGGGGGGGGHNNGGGFDGMGSPAPSNGGDDVYEVHDNHLKAPVSVRREDV 159

Tb9  IPTTGSRGLFNCAQLPSNGNSGGNCNNNSNSNGNGGKSFARICDAHPEGRCISGEQCPDIHVQAEYLTAQRQEMIFWLES 183
Tco  FPTAGSRALFNPQQR--NNGAGG--NGN-----VGGRNFIRICDLFQEGRCLSGALCLDVHVRSDYLNSQRQEMLRWLAS 175
Tv   FPTLGSFGLFRSSQN----GANN----N-----AGGRSTARICDLFHEGRCASSESCSDIHVHPEYLVSKRQEMFSWLEN 169
TcM  SPSVGSRLLFSSQHP----TAG-----------PGSRSVVRVCENFQEGHCVLGENCNDIHLHPEYLMAKRQAMISWIHS 167
Lmj  VSTLGSARLFSKPPK------P---------IVYGSRCVVRICDAFQEGRCAAGDRCHDIHVRPEYLAETRMEMSSWLFD 195
Emt  VATVGSTRLFSKPPK------P---------IVYGSRSVVRICDAFQEGRCASGERCHDIHVKPDYLTQTRLEMSSWLCD 205
Cf   VPTIGSNRLFSRPPK------A---------IVYGSRSVVRICDAFQEGRCLAGEKCHDIHVRPEFLADTRMQMSSWLMD 222
Ang  IATAGSQRLFSNGTR------Q---------IVYGSKNMFRICENFQEGNCLSAN-CPEVHVNPEYLKQTRQALTTWLQE 207
Bs   EETIGSQRLFSAPAQQLNSATAT----------GPQRNAPRICEPYQQGQCMSGDECPDVHVDVNVLQGIRSQMVTWLNA 229

Tb9  KEREFQNTLRADPTKMFRVFCADLKEAVEVPISALQFTKGLYVDPSVRARRARAGHQNQFAMLASQLPTACGLFLSNPSQ 263
Tco  KEEEFMNTLRVDPERIFRVFCADLKETVDVPIAALQFTKGLYVDPSVRARRARGGHQNQYAMMASQLPTACGLFLMDPSY 255
Tv   SEKEFQNTLDADPNKNFRVFCADLKEIVSVPISALRFTKGLYVDPSVRARRTRGGHQNQYAILASQVPTACGLFLLVPSQ 249
TcM  KEREFQNTLIHDPRKVFRVFCADLKEVVEVPISALRFTKGLYVDPNLRARRARSGHQNQFAVMASQVPTACGLFSEDPSQ 247
Lmj  RECEFRQTMEMDSEKTFSIFVADLKEVVEVPINSLVFTKGLYVDPATRAKRARGAGNHAHVHAMMQVPTSCGLYSTDPLQ 275
Emt  RECEFRKTLETDAEKTFSIFVADLKEVVEVPINSLVFTKGLYVDPTTRAKRARSAGNHSHVHAVMQAPTSCGLYSSDPHQ 285
Cf   RENEFRQTLETDPAKTFRIFVADLKEVVEVPVASLVFTKGLYVDPTTRAKRSRGVGNHAHTHAVMQVPTSCGLYSSDPAQ 302
Ang  KENEFNRDFVHHPDKLLKIFVADVKEIVEVPISALVFTKGLYVDPSTRFKRTRGVAPSYNAAIAAQVPTGCGLYSSDPTQ 287
Bs   KEGEFNNTALEAPDTSFRVFAADLKEVVDVPIAALSFTRGLYVDPSARAKRSRYGQQSAFALMAAQVPTACGLHSVDPAQ 309

Tb9  CRWARWCNQIHVDFTWMQTKKKEFESWFNELEGRFNALPDDYEFHVHDPQLKMGLRIPKASIAVFSRGLFQGSPKKAPSV 343
Tco  CKWGRWCNQIHIEISWMQSKKREFEYWFNGLESRFNDLPPDYEFNVHDPQLKTGLRLPKASIAVFSRGLFQGSLKKAPSV 335
Tv   CKWGRWCNQIHIDAGWMQSMKKEFELWSNKLEVYFNSLPPQYQFTVHDPQLKASLSLPKACISGFSRGLFQGSAKKAPSV 329
TcM  CKWGKWCNQVHIEQGWMQFKKNEFESWSNGLERRFNELRPDHLFSVHDPQLKTTLSLPKASIAGFSRGLFQGSAKKAPSV 327
Lmj  CKWERWCNQVHIDHRWMQVKKAEFDQWFIELQNRYFSLAPDDTFTVHDPQLKSSISLPKFSIAGFSRGLFQGSMKKLASV 355
Emt  CKWERWCNQVHIDHRWMKAKKAEFDEWFIELQNRYFALSPDETFTVHDPKLKCSTVLPKFSIAGFSRGLFQGSAKKLASV 365
Cf   CKWGRWCNQVHIDHQWMQSKKEEFDHWFNELQSRYFNLAPDDNFTVHDPQLKTSISLPKFSIAGFSRGLFQGSMKKLASV 382
Ang  CKWGRWCNQVHISPDWMKSKKKNFDSWFDEIEGTYLSYPPEHLFTVHDPQLKRTLQIPKWSVVSFSRGLFQGSDRKAPSV 367
Bs   CKWGRWCNQAHIDPNWMRNKRGEFENWSKSLEQRFEEMPEEHTFVVHDPQLKASLQVPKMAIAGFSRGLFQGNPKKAPSV 389

Tb9  CMLFQRDRCTANVCCNQIHVIPRYLALHRLLIQKGDSITEEE--RGNIMNQMKEILES--QKRSMQVADDGDANFLAREL 419
Tco  CMLFQRNRCTANVCCNQIHVVPQYLNLYRRLVQRGDSLTEAE--RAELTSEMNMVLESYRQDRPLQGGDGNDVSSIARRL 413
Tv   CMLFQRNRCTANACCNQIHVVPEYLNLRRRLAQNTEMMAEE---RDELVRQMEAILNSIE--CNRGSGVNNDVITAAHKD 404
TcM  CMLFQRSRCTANTCCNQIHVDPSYLQLHRRWVCNGEQLPEEK--RRELWEQMNSVLESLLSRCEREAVATNDMYDPAREL 405
Lmj  CLLFQRGKCTAGSCCNQIHVFPQYLSLAREFAAMEQSPDALPEEKQRLRREMELLRGPLMKQEKQE-------TAAMESA 428
Emt  CLLFQRGKCTAGSCCNQIHVCPQYLNLVRECALMEQSPDAMAHESQRLRHEMELLRAPLIEKQLQEQREAR-QAMAREPL 444
Cf   CLLYQRAKCTAGSCCNQIHVVPQYLSLAREYATLEQSPDAPPEEKQRVAHEMAAMRGPCVEQQLREQQEAERAAAELQAV 462
Ang  CLLFQKSRCTAGACCNQIHVSTHYLNLVRTHANNMAQVATTDGYDPAYKNELDQLMETLTAVQKEE-----------EQK 436
Bs   CMLYQRGRCTAGSCCNQIHVFPEYLVLLRQYTAEPTQELFFTMQQVAMQQKQIFLMQQRQSAIQNAAAASGSLNPGARPF 469
```

```
Tb9 NPQA-----------VPYVPP--QPTS-----------------------VWRTG------GSAGSSSPPRTAAAPI--- 454
Tco NPQA-----------TPYTPQPLSPTTGSP-------------------SVWRSG------NPSDNKERKQTPRS----- 452
Tv  FLSA-----------PASVSAMTSRVDPGN---GMDNCVESIRLGLPLSQGVGEG---TQWQS-DSAPAPSTCPVDA--- 463
TcM NPQA-----------QPFVPSPLSSPQPAEKIMGETGAKRKEKMKVDACQVKGEGKGITSFASRQSRNHFPDYPCST--- 471
Lmj SLPN-----------VVSSANEGTHSDAMD-------------IKAAAEEDLASCGALFEHVQVDEDVEPVSHLVSANTS 484
Emt PYPS-----------VVSLPNKESQESSVD-------------VQATAEDDLGCSGPLFEHVQVDEDVEPVSHTVSANTS 500
Cf  QVYP-----------SDQMASEDVSGGTGN-------------GNGPLEDELGSSGALFEHVQVDEDAEPVSHPVSANTS 518
Ang RRAE-----------REQEKAQKTKEEKKK-------------KEETATRDDGES--LFEYIAVDDEADVETSRSPP--- 487
Bs  VPPGGGGGGMGGGGMMGMPQAMNMAAPGGMFFAPMMGMMGLVPMMMAPQQDPSSATGISPYGSHSVTPQHQVFDQQQQ-- 547

Tb9 ------PVPEPLPTFRQPPQWNEHSPSAAVDGSPYASPHRRVERCGSSGTRDLHTCSNPL-------EDLIEFVD-GDKS 520
Tco ------ASPIRIPSFPPPSVLATRDRIINADNCDHALPPQDIVGRSSPIFRGDVGCSNPS-------EDLVEVVD-GDHS 518
Tv  ------GVKNTFGSTHVWPCSATRTLPVHENHSMDIGASSVGDADGR-LLSSTTVVNEGQ---LDLGADLVTVVE-EEQL 532
TcM ------EIEIVCTPMKSQGMVSTGTRSSQQHHQQQEGSNKNVSANGTNGINSRARNNNSAGAFLPLG-EFVTVVDGDERS 544
Lmj LTRRMNASAPDISLSVARHMNSDGSYSFNPYGSVTSLPDGSTSFNRFPGTRG-SGQPMDGNGSVQYTPGSVQLLSTDTKG 563
Emt LSRRLNTSAPDINLLAARHVNSDGSYSFNPYGSLTSLPEGSTSYNRFPGTRG-SGHAVDGNSTTQFTPGNVQVLATGSKG 579
Cf  LTRGLNASAPDINLSSGRHVNNDGSYSYNPYGSVTSLPDASTSYGRAPMGRGGACVPMESSGVVQYTPGNVQLHSTGVKG 598
Ang -------HADGNSSKAGSKKSAGGSYSNNPYS--GSLTDLLFQN--PIVIGG---VPTERNANSVSSFKRTPAASLTCSG 553
Bs  --Q--QQHRQSPTTTVSPSSVQQQQYPQQHHQQQQQQPHQSSAGNDSFYRKPHHHELGSQEPLVQFTPTHDDVVEEHHRA 623

Tb9 RTSLLAS---------QSTTSQASRSCKHTNNPYAFGASYKCTASPTFGWVQPVY------------------------- 566
Tco YSSHPAS---------QSVSSPNCRSTRHTNNPYAFGGNSRCTASPVFGWVQPIR------------------------- 564
Tv  QSSLQAS---------YTNM-YASKGLRRLNNLCGQPGVATCTASPTFGWVQPVH------------------------- 577
TcM QSSLQAS---------HSNISHAGRSYKHTNNPYTFGGSSSCAASPSFGWVQPIR------------------------- 590
Lmj MRRLVAL---------VSPESGNGAVELHRNGDDMGIGGNAGDGGTATVAHDRAPPFAAGHRSNGGYRAVAHPLGDEGGR 634
Emt IRRLVAL---------VSPELGSSMTEAHRNGEENVLSGNTGDSGFSSGAADMAH-VAAMHRSNSGYRAKAHVSGSDGRR 649
Cf  GRRLVAV---------ISPETSSSMTEVHR-MDEMGTSGTVDGAGPASMMDRHAY-APALLRSGGGG-GGAYRAAMHHR- 665
Ang EVAVTSS---------LSTHHQTSHTTATVQAEEGHPEEGDPLQGRSVTSDERGR------------------------- 599
Bs  SASLSSSPNLGPKSPSASVTSTIRQVLSHSPYKRSNLADLRPDNDEVFDRHRTSVVGSGVGGGGAPQPSAHNSNSGNNAQ 703

Tb9 -----SAANSGSTHPAN----------RSWGSGSVTGGCQPIRV---RVGGGGIMEHEPLEPFSLDQHDMGASPFEC-RS 627
Tco -----SAVNSLTMPPVT----------SSWCNDSGTSNNVPFQG---RTEDDSTAAHEGAQPFSLDRHDLEADTFDCGVV 626
Tv  -----SAGNSITNNPMN----------LSCGGNSEMLSKTSS-----ASPVHSDSCALAMMSPLMGGQHGAPDMFSG-FS 636
TcM -----SAGNSCTTMPN-----------SCGGAENGGSNGGNGVWGMRTKADPSPAAADNRSTLFGAHHEFDNSFCA-LS 652
Lmj YDSGTQGEQSCSLHPLDFDASWHFSPTPLLGFDASTTSTHRNG----SIRDEDPAMHAHLRLTASNSSYIYPGSSTTSQQ 710
Emt CDGVAQGEQSSSLQPLDFDASLHFSPYPLLGFDASTTSTHRNG----SLREEDPFMHAQARQAASTSSYPYPGSSTTSQQ 725
Cf  -DGASAHDQSSSLHPLDFDASLHFSPSPLLNFDASNTSLYRNA----STREEETANSLHMRLAASNTSASYAGNSTNSHP 740
Ang -----------------------------------SVT-------KVGEASATSWTNLRFNFDSSVDTYPSVTNGPIP 635
Bs  QVNLQNSFTSNSASSPMKRNNPYQAAQKPIGVPPSPLHAAMPPPPAQTVSNNSMPHTPPMQFSSWAPPSRMPPSMAIPPS 783

Tb9 P--------------------AHRTLGSRTIE-------DRLSSSPTWSATQKQR 655
Tco P--------------------PTSGMVFHTGE-------DSPGSSPT 646
Tv  P--------------------PSLHLSLTNAS---FRALDCTECVAAESLVAPTY 668
TcM PSFQWNASGSTPAKTIEELQKVSPDMDHANAEENGFLGTPASGSTPAKTIEELQKVS------PDMDHTNAEENGFLETP 726
Lmj N-------------------LLSTPMHRGVTSNATTAARVRLAASAASSRTHAHRS---ATSLPDSSTTNPAMGGSVSSM 768
Emt N-------------------LLFTPMQRGLPSNATGAVKVRLAANAAPPRTHTLRS---ATSLSDAFNTTLSMGGSVS-- 781
Cf  --------------------YMMTPAQQSTALNASKSRYNPPTTAPMRTRPSPAAAGNMSSEIATPPFSHAHMSGSAHSN 800
Ang G-------------------SPLGEITRGADNYSI GFLETP 651
Bs  PFRGPGSSMSN-----LPTSPHQGPHTNFTPDMSHVQFSASPLMQPADSLTHLPALRNLTLDNFDDFHLQQSMHGGSLTH 858

Lmj PTARPTFVGEQLTGTR 784
Emt -GTKLTPTGEPSSGTG 796
Cf  PSARPAVVGDDNSGR 815
Bs  HPGSSALGMSGSGIPPAYQGNGSFSSGPHLAGHAANVRQSQPYHHHSNQ 907
```

CCCH MKT1-binding Phosphorylation

At least 5 match:
Template group (chemical)
Template residue

B. Tethering screen results

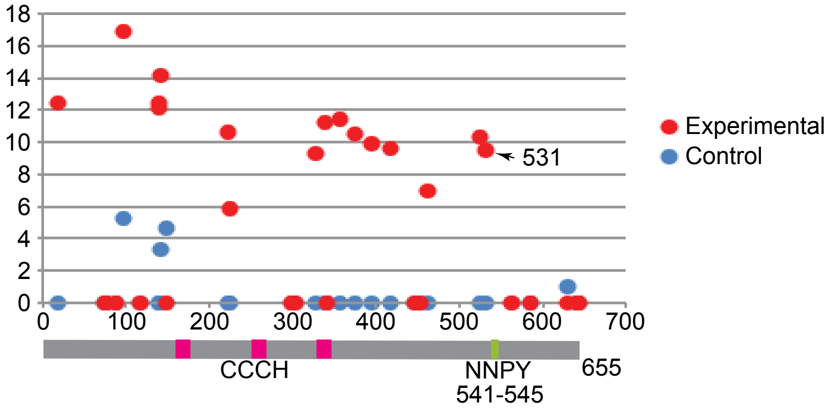

C. Structural predictions

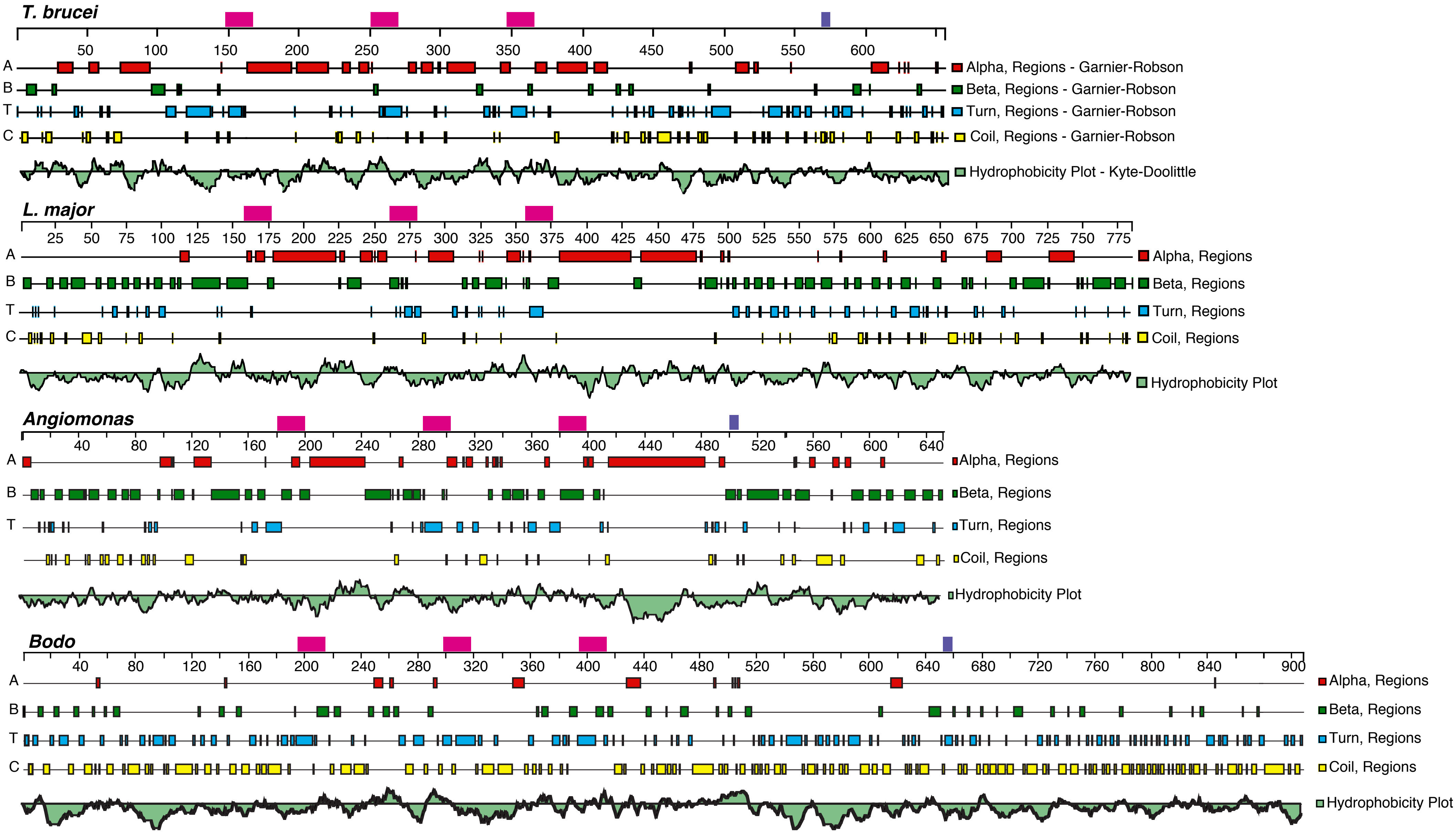

Supplement: S1 Fig — (A) Sequence alignment of ZC3H32 from T. brucei TREU927 (Tb) (XP_822770.1), T. congolense (Tco) (CCC93676.1), T. cruzi (Tc) (EKF99112.1), T. vivax (Tv) (CCC51478.1), Leishmania major (Lmj) (XP_001686656.1), Endotrypanum monterogeii (Emt), Crithidia fasciculata (Cf) (CFAC1_250016000 in TritrypDB), Angomonas deanei (Ang) (EPY19775.1) and Bodo saltans (Bs) (CUF98976.1). The Cx7Cx5Cx3H zinc fingers are indicated with magenta and cyan shading, the Cx9Cx5Cx3H zinc finger is indicated with orange and cyan shading, and the putative MKT1 binding motif (NNPY) with purple shading. Mapped phosphorylation sites in the T. brucei protein [17] are indicated with a red "P". (B) Interactions with MKT1—Mapping of reads in the yeast two-hybrid screen [15]. The blue dots show reads after screening with the empty vector; the red ones are from using MKT as bait. The position of each dot shows the beginning of the peptide encoded by the interacting clone. The NNPY sequence of ZC3H32 starts at position 541, and only sequences that started upstream of this were able to interact with MKT1. (C) ZC3H32 protein secondary structures predicted using DNAStar. The pink segments at the top are the CCCH domains and the blue block is the NNPY. (PDF) [file pone.0177901.s001.pdf]
