# Supplementary figures and images for: The role of the zinc finger protein ZC3H32 in bloodstream-form Trypanosoma brucei
Source: PLoS One. 2017 May 17;12(5):e0177901. doi: 10.1371/journal.pone.0177901 (PMC5435347; doi:10.1371/journal.pone.0177901)

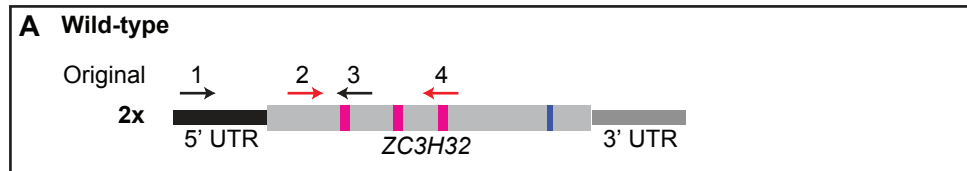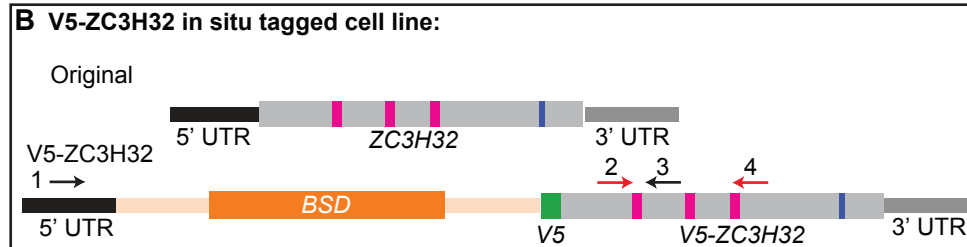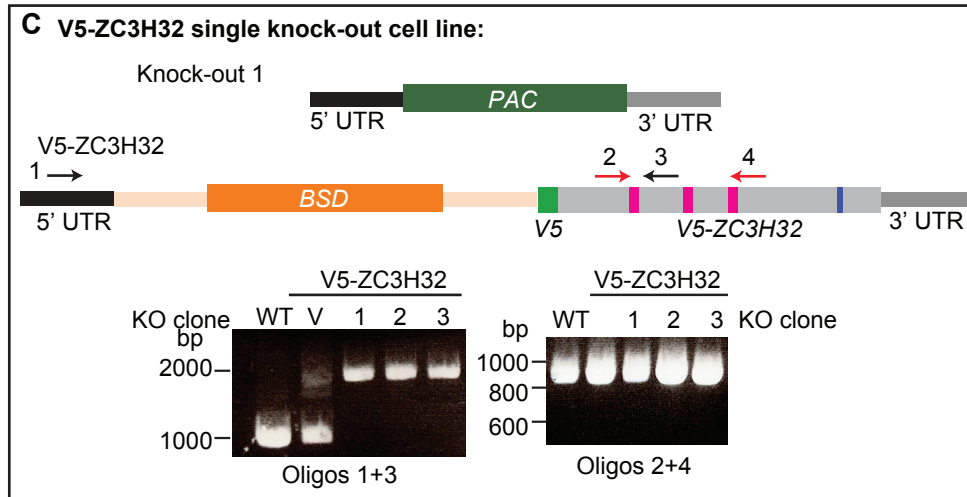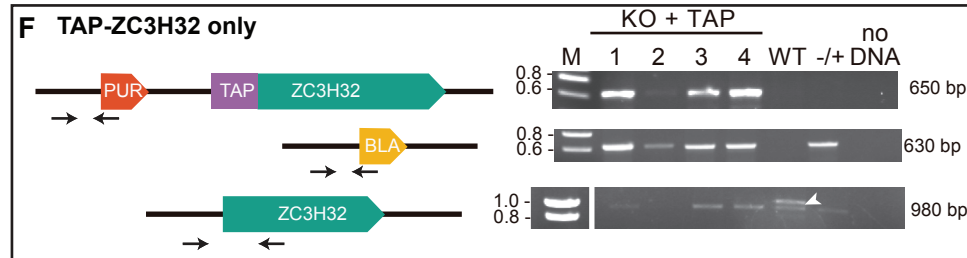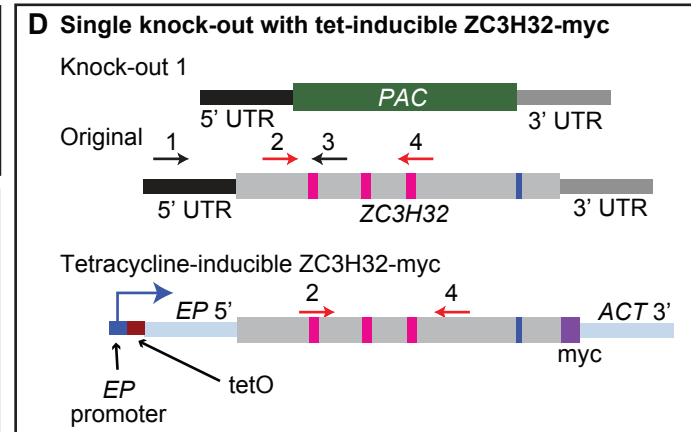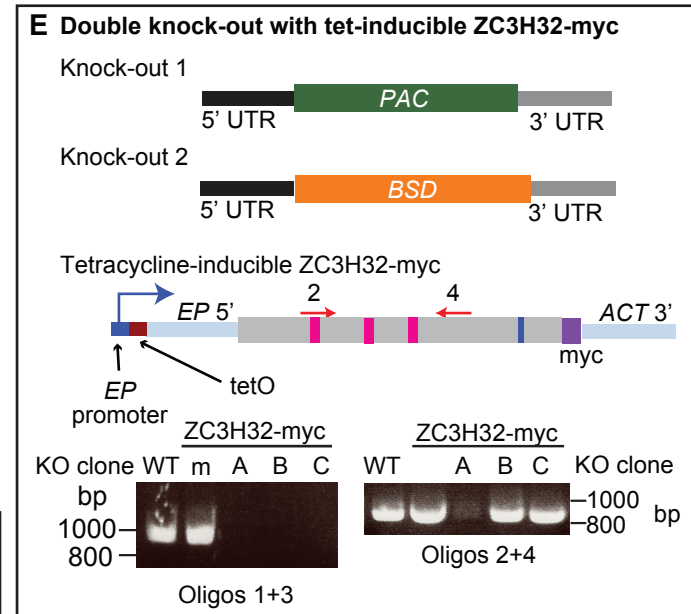

Supplement: S2 Fig — (A) Schematic structure of the ZC3H32 gene. Oligonucleotides used for amplifications are indicated. (B—F) construction of different cell lines, with PCR verification results. WT: wild-type; V: one V5-tagged copy, one normal copy; m: myc-tagged copy without knock-out. BSD: blasticidin S deaminase gene, PAC: puromycin resistance gene. (PDF) [file pone.0177901.s002.pdf]

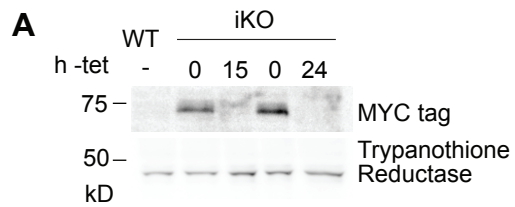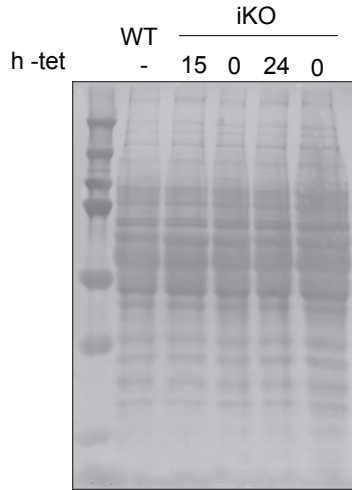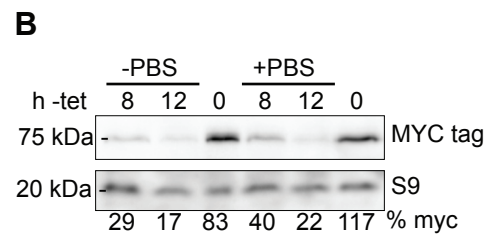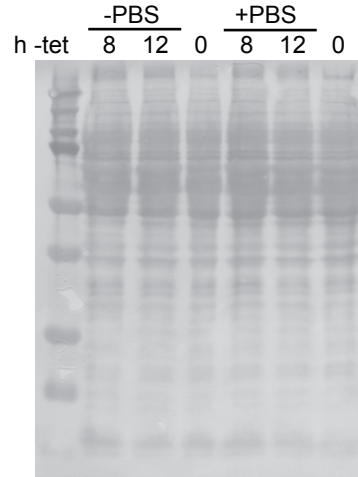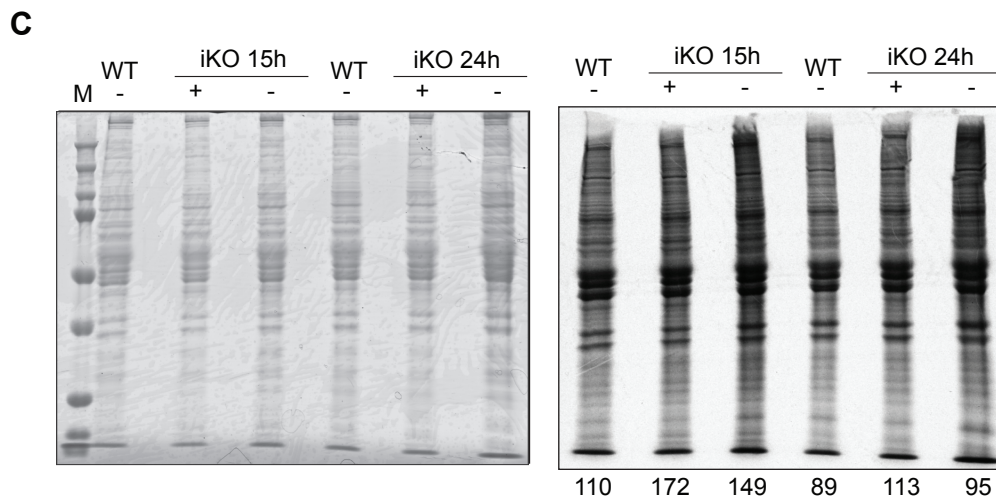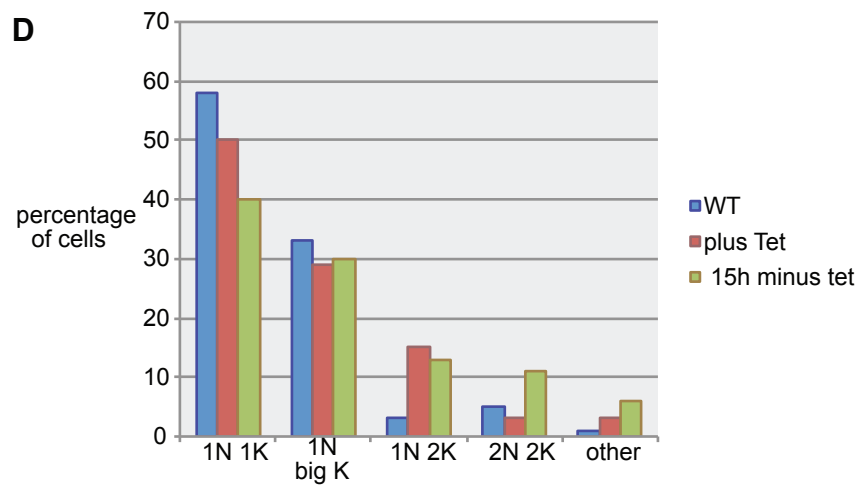

Supplement: S3 Fig — All experiments were done in cells with a single copy of tetracycline-inducible ZC3H32-myc (inducible knock-out or iKO). (A, B) Levels of ZC3H32-myc after tetracycline withdrawal. Cells were centrifuged, as much medium as possible was removed, and then cells were resuspended in tetracycline-free medium, which should reduce the tetracycline concentration to below 5 ng/ml. In panel B we also checked the effect of washing with PBS but this was clearly not necessary. The lower panel is the Ponceau-red stained membrane. (C) Cells were pulsed with [35S]-methionine at various times after tetracycline withdrawal. The Coomassie-stained SDS-PAGE is on the left and the autoradiogram is on the right. For quantification the overall density on each lane of the autoradiogram was normalised to that of the Coomassie stain. The results for experimental lanes were expressed relative to the average for the two wild-type controls, which was set at 100%. (D) Cell cycle stages of trypanosomes after tetracycline withdrawal. The numbers of nuclei (N) and kinetoplasts (K) were counted. 1N1K cells are in G1 phase; 1N-bigK are replicating kinetoplast DNA and will be in S phase; 1N2K is late S or G2; and 2N2K is after mitosis but before cytokinesis. (PDF) [file pone.0177901.s003.pdf]

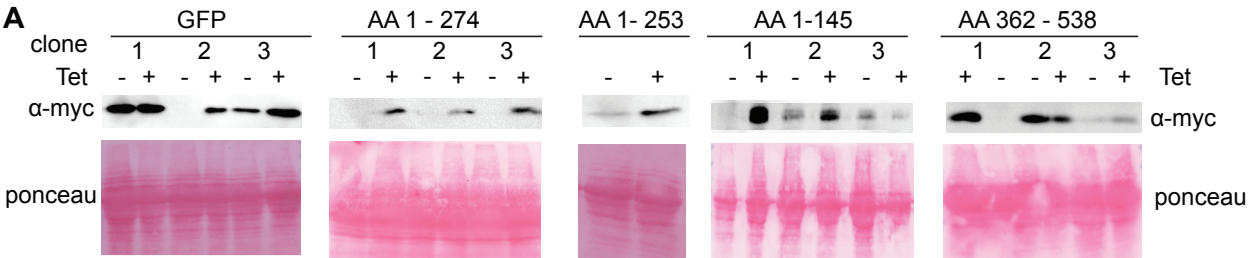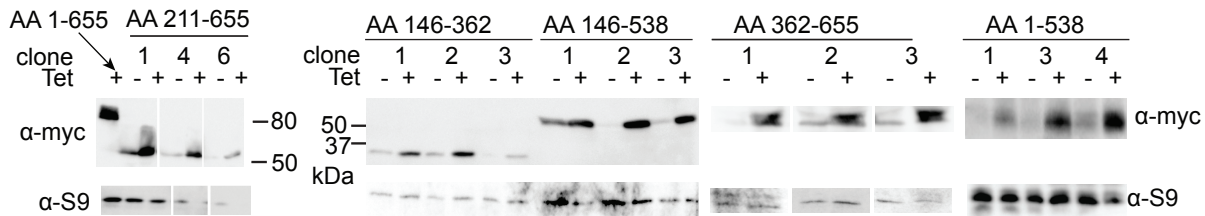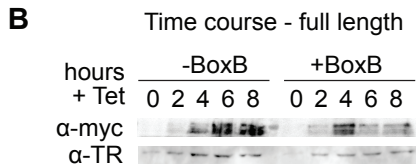

Supplement: S4 Fig — (A) Fragments; the Ponceau stain or ribosomal protein S9 are shown as a controls. (B) Full-length protein. (PDF) [file pone.0177901.s004.pdf]

**A**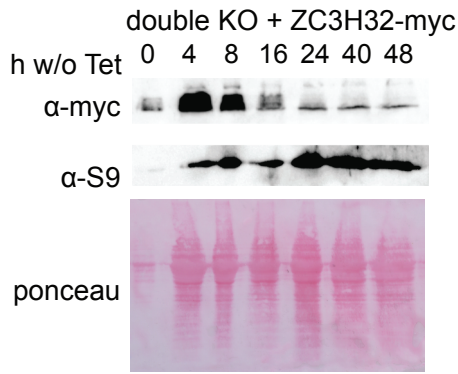**B**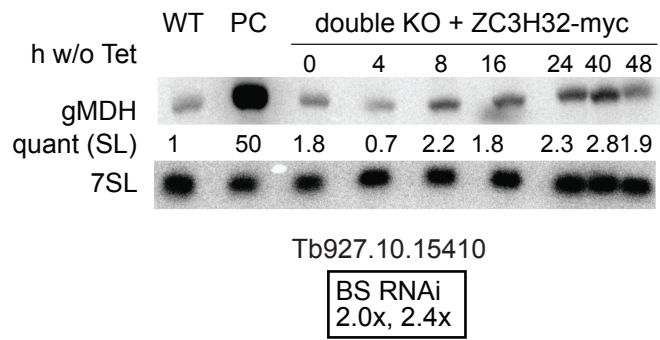**C**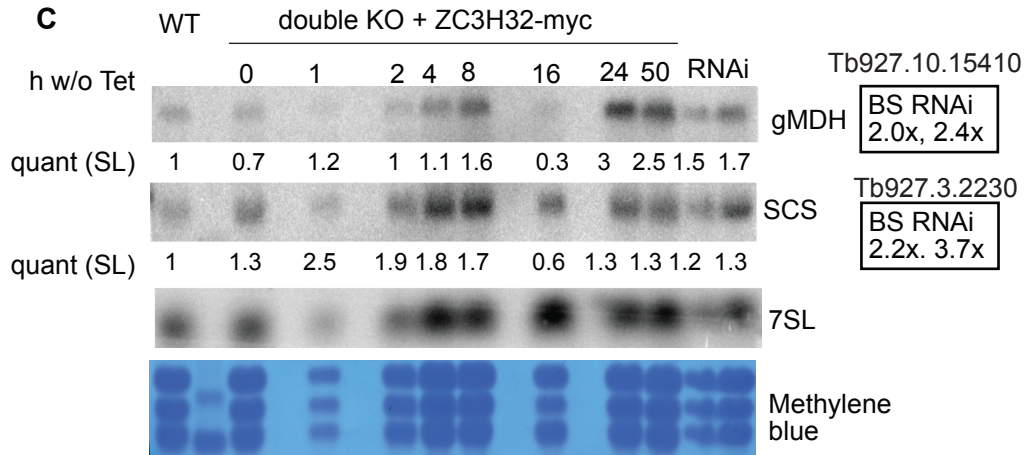**D**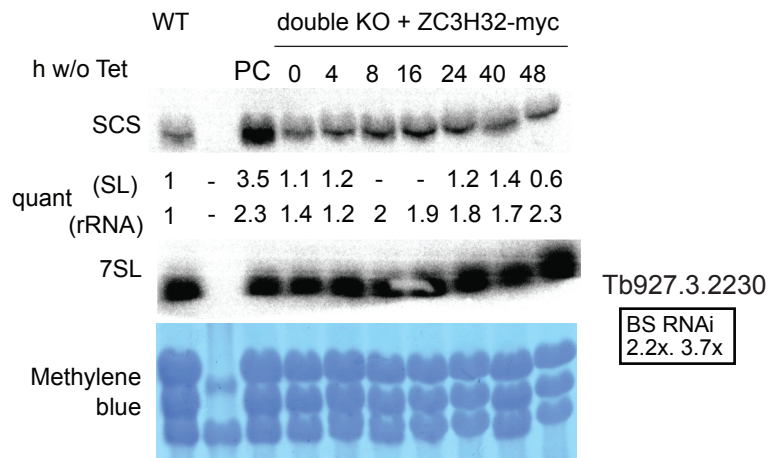**E**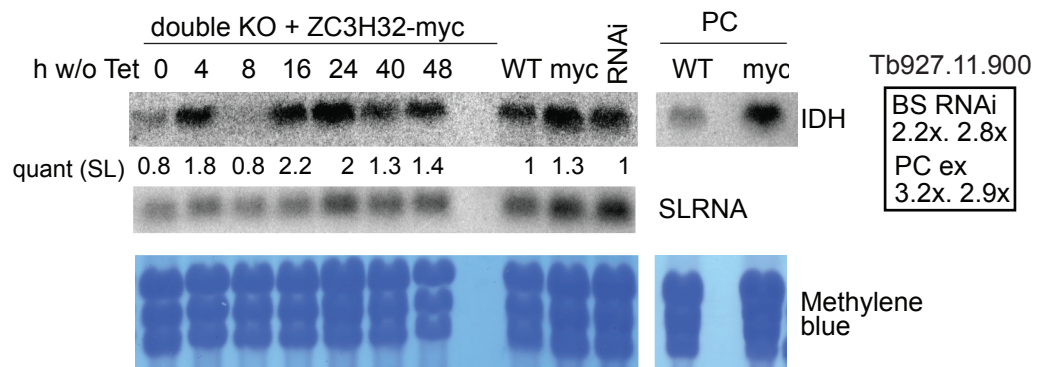

Supplement: S5 Fig — (A) Western blot after tetracycline removal, inducible knock-out cell line. The remaining blots are Northern blots, with quantitation using the 7SL RNA as a loading control. (B) Glycosomal malate dehydrogenase, gMDH. (C) Repeat of gMDH, plus succinyl coA synthetase (alpha subunit); the results from two RNAi experiments are also indicated. (D) Repeat for succinyl coA synthetase. (E) Isocitrate dehydrogenase. (PDF) [file pone.0177901.s005.pdf]
