# Supplementary material for: The role of the zinc finger protein ZC3H32 in bloodstream-form Trypanosoma brucei
Source: PLoS One. 2017 May 17;12(5):e0177901. doi: 10.1371/journal.pone.0177901 (PMC5435347; doi:10.1371/journal.pone.0177901)
Supplement: S4 Table — (DOCX) [file pone.0177901.s009.docx]

### S4 Plasmids and oligonucleotides

**S4A: plasmids**

**Previously existing constructs**

| **pHD** | **description** | **reference** |
| --- | --- | --- |
| 1747 | Blasticidin resistance gene in pBS, for knock-out constructs |  |
| 1748 | Puromycin resistance gene in pBS for knock-out constructs |  |
| p2T7 | Vector with opposing inducible T7 promoters | {Alibu, 2004 #646} |
| 2412 | Tetracycline-inducible expression of lambdaN-any_protein-myc with polylinker with MluI, MfeI, HpaI and BamHI sites | {Singh, 2014 #2791} |
| 1700 | Tetracycline-inducible expression of protein with 2 C-terminal myc tags | {Maier, 2001 #352} |
| p2676 | N-terminal *in situ* TAP tagging vector | {Kelly, 2007 #1488} |
|  | Vector for in situ V5 tagging | {Shen, 2001 #651} |
| 1995 | MKT1 fragment with N-terminal in situ YFP-tag | {Singh, 2014 #2791} |
| 2277 | CAT reporter for tethering, *EP* 5'-UTR, 6x boxB before actin 3'UTR | {Wurst, 2012 #2476} |
| 1991 | CAT reporter for tethering, EP 5'UTR, actin 3'UTR | {Wurst, 2012 #2476} |

**ZC3H32 constructs**

| **pHD** | **description** | **purpose** |
| --- | --- | --- |
| 2332 | pHD1700 + ZC3H32 ORF | inducible expression of ZC3H32-myc |
| 2343 | Bla-V5 ZC3H32 ORF +UTR | in situ tagging of *ZC3H32* with N-terminal V5 |
| 2375 | P2T7TABlue ZC3H32 | T7-driven *ZC3H32* RNAi |
| 2487 | ZC3H32 KO - in 1748 | Knock-out, blasticidin resistance |
| 2488 | ZC3H32 KO - in 1747 | Knock-out, puromycin resistance |
| 2413 | pHD2412 + ZC3H32 | Tethering |
| 2582 | pHD2412 + ZC3H32 AA 1-145 | Tethering |
| 2439 | pHd2412 + ZC3H32 AA 1-158 | Tethering |
| 2482 | pHD2412 + ZC3H32 AA 1-253 | Tethering |
| 2581 | pHD2412 + ZC3H32 AA 1-253 | Tethering |
| 2483 | pHD2412 + ZC3H32 AA 1-274 | Tethering |
| 2484 | pHD2412 + ZC3H32 AA 1-362 | Tethering |
| 2437 | pHD2412 + ZC3H32 AA 83-655 | Tethering |
| 2623 | pHD2412 + ZC3H32 AA 146-551 | Tethering |
| 2594 | pHD2412 + ZC3H32 AA 146-362 | Tethering |
| 2595 | pHD2412 + ZC3H32 AA 146-538 | Tethering |
| 2438 | pHD2412 + ZC3H32 AA 211-655 | Tethering |
| 2485 | pHD2412 + ZC3H32 AA 274-655 | Tethering |
| 2486 | pHD2412 + ZC3H32 AA 362-655 | Tethering |
| 2580 | pHD2412 + ZC3H32 AA 362-538 | Tethering |
| 2622 | pHD 2412 + ZC3H32 AA 529-655 | Tethering |
| 2874 | p2676 + ZC3H32 ORF 5' + 5'UTR | N-terminal *in situ* TAP in the ZC3H32 locus |

**S4B: Oligonucleotides**

| **CZ number** | **name** | **sequence** | **used for**  **pHD** |
| --- | --- | --- | --- |
| 4087 | ZC3H32Hpa1rvb | TATgttaacTCTCTGTTTCTGCGTGGC | 2332  2413 2437 2438 2485 2486 |
| 4501 | ZC3H32Hind3fw | GCGaagcttATGTCTGGCACTAATCAC | 2332 |
| 4678 | Z32UTRRvXbaI | GCGtctagaGATGTTTTCCTTCCCCTT | 2343 |
| 4679 | Z32UTRfwSac2 | CATccgcggACTCACTCATACATCTAA | 2343 |
| 4681 | Z32ORFrvApa1 | ATAgggcccAAGCCAGAATATCATCT | 2343 |
| 4682 | Z32ORFfwXho1 | CATctcgagATGTCTGGCACTAATCAC | 2343 |
| 4747 | ZC3H32 RNAi fw | GAAGAAGAGGACGCTGATGG | 2375 |
| 4748 | ZC3H32 primer rv | ACTGCGAAGGGTTGCTAAGA | 2375 |
| 4978 | Z32.83.fw.asc1 | TATGGCGCGCCtaTCTTTTGAGGTTCAC | 2437 |
| 4979 | Z32.211.fw.asc1 | TATGGCGCGCCtaGTGGAAGTTCCGATT | 2438 |
| 4980 | Z32-538-rvHpa1 | CATgttaacGCAACTGCGACTAGCCTGACT | 2439 2580 2595 |
| 5069 | Z32 KO rv Sac2 3’UTR | ataCCGCGGtcctgacttggttcgtta | 2487 2488 |
| 5070 | Z32 KO fw Xho1 5’UTR | tatCTCGAGttcaggaaattgtgtcgt | 2487  2488 |
| 5071 | Z32 KO rv hind 3 5’UTR | ataAAGCTTgatgttttccttccccttc | 2487 2488 |
| 5072 | Z32 KO fw Xba1 3’UTR | cgcTCTAGAttgtgcctcgttaccatggt | 2487 2488 |
| 5073 | Z32.274.fw.asc1 | TATGGCGCGCCtaGTCGACTTCACATGG | 2485 |
| 5074 | Z32.274.rvHpa1 | GAGgttaacCTGCATCCATGTGAAGTCGAC | 2483 |
| 5075 | Z32teth253rvHpa1 | GAGgttaacGGCAGTTGGTAACTGCGA | 2581 |
| 5076 | Z32teth362rvHpa1 | GAGgttaacAGCTAAGTACCGCGGTATCAC | 2484 2594 |
| 5077 | Z32teth362fwAsc1 | TATGGCGCGCCtaGTGATACCGCGGTAC | 2486 2580 |
| 5148 | Z32gatewayfw | AAAGCAGGCTCCATGtctggcactaatcac | 2507 |
| 5149 | Z32 gateway rv | GTACAAGAAAGCTGGGTTtcatctctgtttctgcgt | 2507 |
| 5460 | Z32teth145rvHpa1 | CTCgttaacAATTCGCGCGAAACTTTTCCC | 2582 |
| 5491 | Z32tethfw146ASC1 | TATGGCGCGCCtaTGTGACGCCCATCCC | 2595 2594 |
| 4906 | Z32ASC1tethfw | tat ggc gcg cct atc tgg cac taa tca | 2413  2439 2483 2484 2581 2582 |
